# Measurement and explanation of socioeconomic inequality in catastrophic health care expenditure: evidence from the rural areas of Shaanxi Province

**DOI:** 10.1186/s12913-015-0892-2

**Published:** 2015-07-03

**Authors:** Yongjian Xu, Jianmin Gao, Zhongliang Zhou, Qinxiang Xue, Jinjuan Yang, Hao Luo, Yanli Li, Sha Lai, Gang Chen

**Affiliations:** School of Public Health, Health Science Center, Xi’an Jiaotong University, P.O Box 86, No. 76 West Yanta Road, Xi’an, Shaanxi 710061 China; School of Public Policy and Administration, Xi’an Jiaotong University, Xi’an, China; School of Biological Sciences and Biotechnology, Murdoch University, Perth, Australia; School of Medicine, Flinders University, Adelaide, Australia

**Keywords:** Catastrophic health care expenditure, Out-of-pocket, Decomposition, Socioeconomic inequality

## Abstract

**Background:**

Policy interventions have been taken to protect households from facing unpredictable economic changes that may cause catastrophe in China. This study aims to estimate the change of overall proportion of households incurring catastrophic health care expenditure (CHE) and its income-related inequality in the rural areas of Shaanxi Province from 2008 to 2013.

**Methods:**

The data were drawn from the National Household Health Service Surveys of Shaanxi Province conducted in the years 2008 and 2013. In total, 3,217 households in 2008 and 13,085 households in 2013 were selected for analysis. A “Capacity to pay” approach was used to measure the incidence of CHE. The concentration index was employed to measure the extent of income-related inequality in CHE. A decomposition method, based on a logit model, was used to decompose the concentration index into its determining components.

**Results:**

From 2008 to 2013, the overall proportion of households incurring CHE dropped from 17.19 % to 15.83 %, while conversely, the inequality in facing CHE strongly increased. The majority of observed inequalities in CHE were explained by household economic status and household size in 2013. In addition, the absence of commercial health insurance and having elderly members were also important contributors to inequality in CHE.

**Conclusions:**

Even though we used a conservative method to measure CHE, the overall proportion of households incurring CHE in Shaanxi Province is still considerably high in both years. Furthermore, there exists a strong pro-rich inequality of CHE in rural areas of Shaanxi Province. Our study suggests that narrowing the gap of household economic status, improving the anti-risk capability of small scale households, establishing prepayment mechanisms in health insurance, strengthening the depth of reimbursement and subsidising vulnerable households in Shaanxi Province are helpful for both reducing the probability of incurring CHE and the pro-rich inequality in CHE.

## Background

Although the Chinese health care system has initiated several reforms designed to eliminate access barriers to health services, millions of people are still prevented from seeking and obtaining needed health care owing to financial difficulty [1]. This is especially true in western China where the per capita income is far behind the eastern areas of China [2]. On the other side of the coin, people accessing health services may cause households to have no choice but to pay a large proportion of household effective earnings, then pushing households into financial hardship or even poverty [3, 4]. Catastrophic health care expenditure (CHE) is a general term used to describe all kinds of health expenditures that pose a threat to the financial capacity of a household in order to maintain its subsistence needs [5–8]. The World Health Organization (WHO) suggested that CHE occurs if out-of -pocket payments (OOP) are at or exceed 40 % of income remaining after household subsistence needs have been met in any year [9]. CHE which could occur in both rich and poor areas is not always a byword of large health care spending [10]. Lacking social support, even a small amount of health spending may be catastrophic to poor households. CHE could force households and their family members to cut back on other consumption, sell assets, make an overdraft on future life, and even be trapped in a long-term debt [11]. Considering the serious consequences of CHE, protecting households from CHE has been widely considered as a desirable objective of many health policies [12–15].

In the past few decades, many policy interventions aimed at reducing OOP health expenditures have been carried out to protect households against CHE in China. One of the most important interventions taken by the Chinese government is to take concrete steps towards the achievement of universal health insurance coverage. Three social health insurance schemes have been implemented in China: the Urban Employee Basic Medical Insurance (UEBMI) designed for the employed urban residents, the Urban Resident Basic Medical Insurance (URBMI) designed for urban residents without formal employment, and the New Rural Cooperative Medical Insurance (NRCMI) designed for rural residents [16, 17]. Take the NRCMI, which was piloted in 2003 and comprehensively implemented in 2008, as an example, by 2013 over 800 million rural residents have participated in NRCMI in rural China. Per capita financing for the NRCMI amounts to around 340 yuan (RMB) (US$ 54.84, all US$ equivalents presented in this paper are calculated based on World Bank annual average exchange rate US$ 1 = RMB 6.20 in 2013), of which 280 yuan (82 %) is subsidised by central and local governments [18]. The actual reimbursement ratio, however, is still at a relatively low level [19]. Other interventions, such as promoting free health treatments to a targeted population, have also been carried out since 2009. By the year 2011, over 39 million elderly people over 65 years old had received health check-ups for free, and over 13 million pregnant and maternal women in the rural areas had received maternity allowance [20].

Despite the fact that “average” catastrophic health spending could be reduced with policy interventions, inequalities in CHE will not simply be eliminated and inevitably exist across households due to geographic and economic factors. Therefore, measurement and regular monitoring of inequalities in CHE are of great concern for policy-decisions, epidemiologists and other health scientists [21].

Previously studies mostly focused on incidence and intensity of CHE and its determinants based upon one-wave cross-sectional survey data [4, 22]. So far, no study has been published to investigate the change of income-related inequality in CHE in western China along with the recent health insurance reform. Considering the unique social structure and economic profiles in local areas, the contributions to inequality in CHE may vary widely between provinces and countries.

The purpose of this study is to measure the overall proportion of households incurring CHE in 2008 and 2013 in Shaanxi rural areas, to compare the change of income-related inequality in CHE between two stages, and to analyse the contributions of determinants to socioeconomic inequality in 2013. The findings of this study will provide readers vital information about the severity of CHE and its inequality in Shaanxi Province. The results will also shed lights on policy suggestions regarding how to reduce the incidence of CHE and its inequality in developing countries.

## Methods

### Data source

Data were drawn from the fourth and fifth Household Health Service Surveys of Shaanxi Province, which were part of China’s National Health Service Surveys (NHSS), conducted in June of 2008 and September of 2013, respectively. NHSS is a national representative survey organised and directed by the Ministry of Health of China every 5 years [23]. Shaanxi Province is located in the northwest of China. With a population of 37.6 million in an area of over 205,800 km^2^, 48.7 % of residents in Shaanxi Province lived in rural areas. The per capital Gross Regional Product (GRP) was RMB 42,692 (US$ 6886) at the end of 2013, lower than that in the eastern areas of China where per capital GRP was RMB 57,722 (US$ 9310).

To achieve representation of the whole population, a four-stage, stratified, random sampling method was adopted in both years. In the first stage, 44 counties (districts) in 2008 and 32 counties (districts) in 2013 were randomly selected in Shaanxi Province. The second stage sampled townships: 75 townships in 2008 and 160 townships in 2013 were selected in sampled counties or districts. In the third stage, 150 villages (communities) in 2008 and 320 villages (communities) in 2013 were selected in sampled townships. In the fourth stage, 5,960 households in 2008 and 20,700 households in 2013 were identified [4].

For each household, a face-to-face interview was conducted using a structured household questionnaire which was developed by the Center for Health Statistics and Information of the Ministry of Health of China. The contents of the household questionnaire included general information on socio-economic and demographic characteristics of the households; demographic and insurance characteristics of household members; self-reported illness and injury; outpatient and inpatient health service utilisation. It is worth mentioning that nine questions were preselected in the questionnaire to measure annual household expenditure, such as food, accommodation, transportation, and OOP health expenditure. The recall period for household expenditure questions was one year prior to the surveys [24]. An adult who was fully aware of the household income and expenditure information was eligible to respond in this section. Information about infants and young children was collected through their parents or guardians. Missing households which were not available after 3 contacts were substituted by alternative households based on predefined protocol.

Considerable quality assurance measures were implemented during the process of data collection. Survey supervisors revisited 5 % of the sampled households to check the accuracy of data recorded by interviewers. In this process, 14 key questions were asked again to check the consistency of the information recorded. The consistency rates of the key questions recorded between the first and second visits was over 95 %. The Myer’s Blended index was 1.67 in 2008 and 1.62 in 2013, indicating that in both years there was no significant difference between the sampled age distribution and the overall age distribution of Shaanxi Province (i.e. non-existence of age preference in the survey) [25].

Data collected from rural areas were used in this study, including 3,239 households in 2008 and 13,200 households in 2013. After data cleaning (i.e. excluding households with logic error answers and/or with key variables missing), 3,217 households in 2008 and 13,085 households in 2013 were adopted for empirical analysis.

### Ethical considerations

The Ethics Committee of Xi’an Jiaotong University Health Science Center approved this study (approval number 2014–204), which conformed to the ethics guidelines of the Declaration of Helsinki. Informed consent was obtained by surveyors prior to data collection.

### Statistical analysis

#### Catastrophic health care expenditure

Although there is no consensus on defining CHE, it is widely agreed that CHE should be measured in relation to a household’s capacity to pay (CTP) [10]. CTP is defined as non-subsistence expenditure, which calculated as total household expenditure minus spending on basic necessities. Generally we use the households’ average food expenditure in the 45^th^ to 55^th^ percentile adjusted for the household scale as a proxy measure for basic necessities [26]. Referring to several WHO studies, we set the catastrophic threshold at 40 % of the household’s CTP [9]. In this study, we used OOP as a numerator and CTP as the denominator to calculate the CHE incurrence. OOP includes all types of direct health-related expenditures made by households, primarily for the purchase of medications (including self-medication), payments for outpatient and/or inpatient care, preventive care, maternal and child health services. A binary variable was defined indicating whether the household incurred CHE or not.

#### Methods to measure CHE inequality

Concentration index, which is one of the most widely accepted methods of defining health inequalities, was employed to measure the extent of socioeconomic inequality in CHE. It is defined as twice the area between the concentration curve and the line of equality [27, 28]. The concentration index lies in the interval [−1, 1] [29]. Its positive value indicates that a variable is more concentrated among the advantaged, and vice versa. The larger the absolute value of concentration index, the greater the inequality in CHE [30]. The formula for computing the concentration index is:

1 $$ \mathrm{C}=\frac{2}{\mu }cov\left({y}_i,{R}_i\right) $$

Where C stands for concentration index, y_i_ is CHE index, μ is the mean of CHE index, and R_i_ is the fractional rank of household in the income distribution.

#### Decomposition methods

Inequality can be further explained by decomposing the concentration index into its determining components. Decomposition methods can enable researchers to quantify each determinant’s true contribution to measured income-related inequality with the controlling of other determinants. When health outcome is continuous, OLS regression is widely used to decompose the concentration index into the contributions of different factors [31]. The formula for the concentration index decomposition using OLS regression can be written as follows:

2 $$ {y}_i=\alpha +{\displaystyle {\sum}_k}{\beta}_{\mathrm{k}}{x}_{k_i}+{\varepsilon}_i $$

where ε is an error term. The concentration index for y(C) can also be written as:

3 $$ C={\displaystyle \sum_k\left({\beta}_k{\overline{x}}_k/\mu \right)}{C}_k+G{C}_{\varepsilon }/\mu $$

Where μ is the mean of the dependent variable, ${\overline{x}}_k$ is the mean of independent variable *x*_*k*_, *C*_*k*_ is the concentration index for *x*_*k*_, and *GC*_*ε*_ is the generalised concentration index for the error term. ${\beta}_k{\overline{x}}_k$ is the elasticity of the dependent variable on the corresponding independent variable. We can see from Equation 3 that the overall inequality has two components: “explained” component captured by the first term and residual or “unexplained” component captured by the last term [32, 33]. However, the OLS regression based estimation fails to deal with cases where the health outcome is binary [19]. To tackle the disadvantage, Hosseinpoor et al. modified this approach to deal with binary outcomes in 2006 [20]. The extension for the decomposition method provides us an opportunity for further analysis on unraveling and quantifying each determinant contribution to socioeconomic inequality in CHE. Following Hosseinpoor et al., we used a non-linear logit model instead of OLS regression to conduct the decomposition analysis. As the logit model is essentially non-linear in the probability of incurring CHE, the natural logarithm of the odds of CHE was used as the dependent variable (rather than actual CHE) for decomposition [34].

4 $$ Ln\left( odd{s}_{CHE}\right)=\alpha {}_i+{\displaystyle \sum {\beta}_i{x}_i+{\varepsilon}_i} $$

All of the analyses were performed in STATA software version 10.0.

### Independent variables

With reference to previous studies, four groups of factors, which may be associated with the CHE were used in this study. Firstly, demographic characteristics include five variables: having elderly members, having children in the household, household size, household head’s gender and educational achievement. Having elderly members is a dummy variable indicating whether there were members in the household 65 years or older. Having children in the household is a dummy variable indicating whether there were members in the household below 5 years old. The second group, illness and treatment history, includes three dummy variables: having chronic disease members (i.e. whether any household member had doctor-diagnosed chronic diseases in the past six months), inpatient service usage (i.e. whether any household member used inpatient services in the past year) and outpatient service usage (i.e. whether any household member used outpatient services in the past two weeks). Thirdly, health insurance characteristics include two dummy variables: absence of social health insurance and absence of commercial health insurance. Lastly, household economic status is measured by annual self-reported household expenditure in our study. Both self-reported household expenditure and household income data are available in the NHSS data; however, it is suggested that for developing countries expenditure data is a better proxy of household economic status than income data since the latter is likely to be under-reported [35]. Households were ranked according to per-capital household expenditure and grouped into five quintiles.

## Results

### Descriptive analysis

Table 1 shows the summary statistics for independent variables. In 2013, 21.64 % of household heads were female, and 16.85 % of them were illiterate. From 2008 to 2013, the percentage of households having 1–2 household members rose rapidly from 36.21 % to 51.69 %, in line with the demographic changes in Shaanxi rural areas. The percentage of households with all members covered by social health insurance increased sharply from 88.13 % to 97.47 %; households with chronic disease members rose from 32.73 % to 40.89 %. In the year 2013, 21.40 % of households used inpatient health services, while this proportion was just 13.99 % in 2008.

**Table 1:** Description of independent variables in 2008 and 2013

Variables	Description	2008	2013
		*N* = 3217	*N* = 13085
		N(%)	N(%)
Having elderly members			
No*	1 if all household members below 65 years old, 0 otherwise.	2431(75.57)	9498(72.59)
Yes	1 if household having members over 65 years old, 0 otherwise	786(24.43)	3587(27.41)
Having children in the household			
No*	1 if all household members over 5 years old, 0 otherwise.	2645(82.22)	10972(83.85)
Yes	1 if household having members below 5 years old, 0 otherwise	572(17.78)	2113(16.15)
Household size			
1-2 members	1 if 1–2 household members, 0 otherwise	1165(36.21)	6764(51.69)
3-4 members	1 if 3–4 household members, 0 otherwise	1452(45.14)	4909(37.52)
≥5 members*	1 if more than 5 household members, 0 otherwise.	600(18.65)	1412(10.79)
Having chronic disease members			
No*	1 if no household members having doctor-diagnosed chronic diseases, 0 otherwise.	2164(67.27)	7734(59.11)
Yes	1 if having household members having doctor-diagnosed chronic diseases, 0 otherwise	1053(32.73)	5351(40.89)
Household head’s gender			
Female*	1 if the head of household was female, 0 otherwise.	556(17.28)	2832(21.64)
Male	1 if the head of household was male, 0 otherwise	2661(82.72)	10253(78.36)
Household head’s educational achievement			
Illiteracy	1 if the head of household was illiteracy, 0 otherwise	618(19.21)	2205(16.85)
Elementary	1 if household head graduating from elementary school, 0 otherwise	1052(32.70)	4262(32.57)
Middle school	1 if household head graduating from middle school, 0 otherwise	1285(39.95)	5336(40.78)
High school and above*	1 if household head graduating from high school or university, 0 otherwise.	262(8.14)	1282(9.80)
Inpatient service usage			
No*	1 if no household members used outpatient services in the last two weeks, 0 otherwise.	2767(86.01)	10285(78.60)
Yes	1 if having household members used outpatient services in the last two weeks, 0 otherwise	450(13.99)	2800(21.40)
Outpatient service usage			
No*	1 if no household members used inpatient services in the last year, 0 otherwise.	2679(83.28)	10605(81.05)
Yes	1 if household members used inpatient services in the last year, 0 otherwise	538(16.72)	2480(18.95)
Absence of social health insurance			
No*	1 if all household members covered by social health insurance, 0 otherwise.	2835(88.13)	12754(97.47)
Yes	1 if having household members not covered by social health insurance, 0 otherwise	382(11.87)	331(2.53)
Absence of commercial health insurance			
No*	1 if having household members covered by commercial health insurance, 0 otherwise.	463(14.39)	1253(9.58)
Yes	1 if all household members not covered by commercial health insurance, 0 otherwise	2754(85.61)	11832(90.42)
Economic status			
Quintile 1 (poorest)	1 if the 20 % low income households, 0 otherwise	645(20.05)	2617(20.00)
Quintile 2 (poorer)	1 if the 20 % low middle income households, 0 otherwise	643(19.99)	2626(20.07)
Quintile 3 (middle)	1 if the 20 % middle income households, 0 otherwise	640(19.89)	2654(20.28)
Quintile 4 (richer)	1 if the 20 % high middle income households, 0 otherwise	636(19.77)	2581(19.73)
Quintile 5 (richest)*	1 if the 20 % high income households, 0 otherwise.	653(20.30)	2607(19.92)

* Reference groups

### Catastrophic health care expenditure

Table 2 displays the average household OOP health expenditure, average household CTP, and the proportion of households with CHE. In the year 2013, the average OOP expenditure was RMB 3052.88 (US$ 492.40) and the average household CTP was RMB 16567.24 (US$ 2672.14). The poorest households had the highest proportion of CHE occurrence compared to other quintiles. In the year 2008, not only the poorest households but also the richest households had a high proportion of CHE incurrence. The overall proportion of households incurring CHE dropped from 17.19 % in 2008 to 15.83 % in 2013, with a statistically significant difference at the level of α = 0.10 (*χ*^2^ = 3.509, *P* = 0.061).

**Table 2:** Number and proportion of households that incurred CHE

Economic status	2008			2013		
	Average OOP health expenditure (yuan RMB)	Average household CTP (yuan RMB)	No.(%) household with CHE	Average OOP health expenditure (yuan RMB)	Average household CTP (yuan RMB)	No.(%) household with CHE
Quintile 1 (poorest)	626.03	2747.05	117(18.14)	1337.06	5530.64	585(22.35)
Quintile 2 (poorer)	1010.58	4242.29	124(19.28)	1994.95	9555.12	413(15.73)
Quintile 3 (middle)	1178.50	5484.92	97(15.16)	2713.22	13914.49	393(14.81)
Quintile 4 (richer)	1650.81	7749.65	96(15.09)	3467.41	19171.17	345(13.37)
Quintile 5 (richest)	3647.62	13561.46	119(18.22)	5776.32	34831.99	336(12.89)
Total	1620.54	6745.79	553(17.19)	3052.88	16567.24	2072(15.83)

OOP, out-of-pocket; CHE, catastrophic health care expenditure; CTP, capacity to pay

### Associations between CHE and its determinants

Table 3 shows the results of estimated odds ratios and corresponding 95 % confidence intervals (CIs) in the logit regression models. As expected, most variables increased the risk of incurring CHE. Households with elderly members over 65 years old increased the odds of incurring CHE 1.59 times in 2008, and 1.57 times in 2013. Households with 1–2 members were 3.04 times in 2008, and 3.68 times in 2013, more likely to suffer from CHE than households with more than 5 members. Having chronic disease members were 2.48 times in 2008, and 1.77 times in 2013, more likely to face CHE compared to households without chronic disease members. Inpatient health service usage increased the odds of suffering CHE 5.88 times in 2008, and 3.69 times in 2013. Absence of commercial health insurance increased the odds of facing CHE 1.50 times in 2008 and 1.51 times in 2013, respectively. The poorest households had the highest odds of incurring CHE in 2013 (OR: 2.25, 95 % CI: 1.90-2.65). There was no statistically significant association between having children below 5 years old and incurring CHE in both years.

**Table 3:** Adjusted associations between CHE and its determinants

Variables	2008		2013	
	OR	95 % CI	OR	95 % CI
Having elderly members	1.59**	(1.25-2.02)	1.57**	(1.40-1.76)
Having children in the household	1.02	(0.73-1.41)	1.03	(0.86-1.24)
Household size				
1-2 members	3.04**	(2.12-4.36)	3.68**	(2.97-4.55)
3-4 members	1.26	(0.90-1.75)	1.45**	(1.18-1.77)
Male household head	1.15	(0.87-1.52)	1.14*	(1.00-1.30)
Household head’s educational achievement				
Illiteracy	2.20**	(1.36-3.56)	2.18**	(1.74-2.71)
Elementary	1.50	(0.95-2.38)	1.58**	(1.28-1.94)
Middle school	1.15	(0.73-1.83)	1.10	(0.90-1.36)
Having chronic disease members	2.48**	(2.00-3.08)	1.77**	(1.58-1.97)
Inpatient service usage	5.88**	(4.50-7.69)	3.69**	(3.29-4.13)
Outpatient service usage	1.30*	(1.01-1.71)	1.26**	(1.11-1.42)
Absence of basic health insurance	1.28*	(1.02-1.55)	1.38**	(1.21-1.59)
Absence of commercial health insurance	1.50*	(1.04-2.17)	1.51**	(1.21-1.87)
Economic status				
Quintile 1 (poorest)	1.15	(0.81-1.63)	2.25**	(1.90-2.65)
Quintile 2 (poorer)	1.37*	(1.01-1.90)	1.50**	(1.26-1.78)
Quintile 3 (middle)	0.91	(0.65-1.27)	1.44**	(1.21-1.71)
Quintile 4 (richer)	0.87	(0.63-1.22)	1.20*	(1.01-1.42)
Observations	3217		13085	
LR chi2(17)	438.29		1548.88	
P	<0.001		<0.001	
Pseudo R^2^	0.158		0.136	

*P <0.05; **P <0.01

### Socioeconomic inequality in CHE

**Fig. 1:**
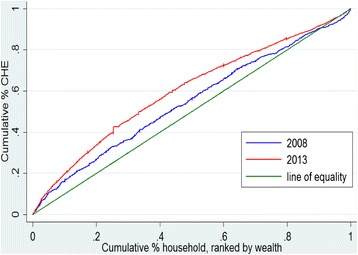
Concentration curves of incurring catastrophic health care expenditure

Figure 1 presents the concentration curves in the years 2008 and 2013. Both concentration curves lay above the 45° line (the line of equality), indicating that the incurring CHE was more concentrated among the poor. From 2008 to 2013, the concentration index in CHE increased substantially from −0.09 (95 % CI: −0.137 to −0.048) to −0.20 (95 % CI: −0.226 to −0.178). Negative concentration indices in both years demonstrate that households with low economic status had a higher probability of incurring CHE than the rich in Shaanxi rural areas. Dominance testing results for concentration curves indicated that there was statistically significant dominance between curves in 2008 and 2013.

### Decomposition of socioeconomic-related inequality

By decomposing the concentration index of the Ln odds _CHE_, the socioeconomic-related inequalities were decomposed into relative contributions of each determinant. Table 4 presents summary results of decomposition in 2013. The second column shows the elasticity of Ln odds _CHE_ with respect to each determinant. The third column shows the concentration index for each determinant. The fourth column shows the pure contributions of determinants to the socioeconomic inequality in Ln odds _CHE_.

**Table 4:** Decomposition analysis of concentration index in CHE in 2013

	Elasticity	Concentration index (C)	Contribution to C	Contribution to C (%)
Having elderly members	−0.062	−0.116	0.007	6.17
Household size				44.11
1-2 family members	−0.338	−0.186	0.063	
3-4 family members	−0.069	0.160	−0.011	
Male household head	−0.051	0.008	0.000	−0.35
Household head’s educational achievement				16.93
Illiteracy	−0.064	−0.256	0.016	
Elementary	−0.075	−0.074	0.006	
Middle school	−0.020	0.107	−0.002	
Having chronic disease members	−0.118	0.021	−0.003	−2.14
Inpatient service usage	−0.142	0.136	−0.019	−16.49
Outpatient service usage	−0.022	0.085	−0.002	−1.61
Absence of social health insurance	−0.003	0.169	−0.001	−0.49
Absence of commercial health insurance	−0.188	−0.031	0.006	4.96
Economic status				48.79
Quintile 1 (poorest)	−0.082	−0.638	0.053	
Quintile 2 (poorer)	−0.041	−0.263	0.011	
Quintile 3 (middle)	−0.037	0.033	−0.001	
Quintile 4 (richer)	−0.018	0.266	−0.005	
C of Ln odds CHE	0.118			
Mean of Ln odds CHE	−1.968			
Observations	13085			

A negative concentration index of determinant variables, such as having elderly members in household and absence of commercial health insurance, means that these variables are more concentrated among the worse off, and vice versa.

The positive value of contribution means that this variable contributes to pro-rich inequality [33]. Similarly, a negative contribution means this relative variable reduces pro-rich inequality. The findings indicated that having elderly members and absence of commercial health insurance increased pro-rich inequality, whereas having chronic disease members, inpatient and outpatient health service usage, and absence of social health insurance reduced the pro-rich inequality, favouring the poor.

Quantifying the corresponding contributions expressed as a percentage of each determinant in 2013, most of the socioeconomic inequality in CHE was attributable to household economic status (48.79 %) and household size (44.11 %).

## Discussion

In the study, we estimated the overall proportion of households incurred CHE between 2008 and 2013 in Shaanxi Province with a conservative method. Firstly, we used a high threshold to define the occurrence of CHE. Secondly, indirect expenditure for seeking health services, such as transportation, food, accommodation, lost earnings due to illness, was not included in health expenditure [9]. This conservative estimation method may lead to underestimating the financial consequences of household health expenditures [9, 36]. Even though a conservative method was used to measure CHE, the proportion of households incurring CHE in Shaanxi Province was still considerably high in both years. This proportion was not only higher than that in most rural areas of other provinces in China, but also higher than that in most developing countries [4, 9, 37, 38]. Several potential factors could be responsible for this phenomenon. Firstly, despite the fact that Shaanxi Province has achieved close to universal health insurance coverage, actual reimbursement ratio for NRCMI designed for rural residents is still at a low level. A high proportion of OOP in the total health expenditure remains a cause of households facing CHE. Secondly, health service access and use is also a very important determinant resulting in CHE. Economic growth generates higher demand for expanded health care provisions. In Shaanxi Province, the outpatient visits ratios within two weeks rose from 11.18 % in 2008 to 12.55 % in 2013, and the annual hospitalisation rate jumped from 5.39 % in 2008 to 10.06 % in 2013 (Yongjian Xu, Yanli Li, Sha Lai, unpublished observations). Increasing usage of health services and the lag of social institution’s development have a potential effect of putting households into economic catastrophe [9]. Thirdly, the long-term split of basic health insurance schemes designed for different target populations—urban and rural, formal and informal—and the financial risk management at county or city-level, lead to the deficiencies of the risk pooling capabilities. Risk pooling shares medical costs with different profiles to prevent households from catastrophic expenditure due to unexpected diseases, and enables cross-subsidies from the advantaged to the disadvantaged [39]. A fourth issue contributing to CHE is that the continuing shrinkage of household size reduces the household’s anti-risk ability to cope with catastrophic payments.

This study emphasised some key factors as determinants of catastrophic health care expenditure. Most were similarly reported in related studies [40, 41]. As we expected, a lower economic status played an important role in increasing the risk of incurring CHE in 2013. However our study found that poorest households showed no statistically significant association with CHE in 2008. One possible explanation is that the poorest households forgo their needed health care due to high health care–related cost. Our unpublished analyses, using the same data, found that in 2008, 36.08 % members in poorest households refused inpatient treatment in the past year, whilst 53.50 % members in these households suffering from illness and injury in the past two weeks chose not to seek medical treatment. Consistent with most previous studies in China, our findings showed that absence of social health insurance increased the risk of households incurring CHE in the rural areas of Shaanxi Province [41]. In addition to household economic status and absence of social health insurance, having elderly members, inpatient and outpatient health services usage, and lack of commercial health insurance were also key determinants of CHE. From 2008 to 2013, the percentage of households having a male head dropped from 82.71 % to 78.36 %, and our study found that the male household head increased the risk of households incurring CHE in 2013, but there was no significant association in 2008. Further studies are needed to explore the reasons. Unlike previous studies conducted in other countries, our study showed that there was no statistical association between CHE and households with a child or children below 5 years old in both years [40]. One of the potential reasons is that an expanded program of immunisation for children, promoted in Shaanxi Province, has successfully prevented and controlled outbreak and spread of many childhood infectious diseases which may make households face large health expenditures [42]. A small household with more inpatient treatments, having elderly members, lack of health insurance and illiteracy of the household head, had the higher risk of incurring CHE. Therefore, policy interventions aimed at reducing the probability of household incurring CHE should primarily consider the needs of vulnerable households.

The issue of CHE will not simply be solved with an increasing trend of income. Local health systems should be improved in several aspects to protect households from CHE. One key approach reducing economic catastrophe is to establish pre-payment mechanisms and move away from post-payment mechanisms in basic health insurance systems [10]. Another approach is to enhance the actual reimbursement ratio of basic insurance (or reduce OOP payment ratios). A third, developing financial risk pooling should be placed on the agenda. In the present circumstances, an incremental approach, which starts with different pools designed for target populations and combines them over time, is a feasible way to risk pooling used by policy-decisions [39].

From 2008 to 2013, local health systems were mainly dedicated to protecting households against catastrophic payments and eliminating access barriers to needed health care by carrying forward universal health coverage as well as other measures. However, it is worth noting that the inequality in facing CHE significantly increased over these years. Thus, further policy interventions should be taken to reform health systems to address the remaining inequality.

Our study revealed that some determinants, such as absence of commercial health insurance and households having elderly members, showed positive contributions to socioeconomic inequality in CHE, which meant CHE was greater among the poor. Furthermore, our study also indicated that households with chronic disease members had negative contributions, favouring the poor. A potential explanation is that most chronic diseases, such as hypertension, diabetes and coronary artery disease, is more prevalent among the advantaged and associated with a high risk of incurring CHE. Consistent with earlier studies, our study found that the contributions of health service usage variables to the probability of incurring CHE were in a pro-poor direction, reducing socioeconomic inequality in 2013 [36, 43]. That is because the rich households are relatively more prone to use these services and accessing such services has a higher risk of incurring CHE, while conversely, poor households are less likely to be affected with little use of health services. From the decomposition analysis of concentration index, it is evident that most of the inequality in CHE is explained by household size and household economic status in 2013. Therefore, potential solutions to relieve socioeconomic inequalities should concern the degree of determinants’ contribution, and it is helpful to reduce inequalities in CHE by narrowing the gap of household economic status and improving anti-risk capability of small scale households.

There are some limitations in the study. Firstly, all of the demographic, socioeconomic and health services usage information were self-reported. Household health expenditure and health services usage data may be less accurate than medical records. Secondly, the presence of missing data may lead to biased estimates of regression parameters and weaken generalisability of our results. However, considering less than 1 % data were excluded owing to the missing value, this problem may not be serious. Thirdly, the way we calculated CHE has some limitations. Poor households choosing not to seek health care were excluded from CHE calculations. Also, since the economic status was measured by annual household expenditure, poor households spending catastrophic expenditure on health services increased their CTP and total expenditure, thus these households were categorised into a higher economic status in analyses. Fourthly, there was likely to be recall bias because of a long recall period.

## Conclusions

Even though we used a conservative method to measure CHE, the overall proportion of households incurring CHE is still considerably high compared to other rural areas of China and most developing countries in the world. Furthermore, there exists a strong pro-rich inequality of CHE in the rural areas of Shaanxi Province. Many determinants, such as absence of health insurance, having elderly members and lower economic status, increased the probability of incurring CHE. Lower economic status and small household size were the main determinants contributing to inequality favouring the rich. This study suggests that in Shaanxi Province, which has almost achieved universal health insurance coverage, narrowing the gap of household economic status, establishing prepayment mechanisms in health insurance, strengthening the depth of reimbursement (reducing OOP ratio) and subsidising vulnerable households are helpful for both reducing the probability of incurring CHE and pro-rich inequality in Shaanxi Province.

## References

[1] Hajizadeh M, Nghiem HS (2011). Out-of-pocket expenditures for hospital care in Iran: who is at risk of incurring catastrophic payments?. Int J Health Care Finance Econ.

[2] Chen G, Inder B, Hollingsworth B (2014). Health investment and economic output in regional China. Contemp Econ Policy.

[3] Xu K, Evans DB, Kawabata K, Zeramdini R, Klavus J, Murray CJ (2003). Household catastrophic health expenditure: a multicountry analysis. Lancet.

[4] Zhou Z, Gao J (2011). Study of catastrophic health expenditure in China’s basic health insurance. HealthMED.

[5] WP SE, Aizuddin AN, Zainuddin Z, Manaf MRA, Aljunid S (2012). Catastrophic health expenditure and its influencing factors in Malaysia. BMC Health Serv Res.

[6] Berki SE (1986). A look at catastrophic medical expenses and the poor. Health Affair.

[7] Wyszewianski L (1986). Families with catastrophic health care expenditures. Health Serv Res.

[8] Wagstaff A, Van Doorslaer E (2002). Catastrophe and impoverishment in paying for health care: with applications to Vietnam 1993–1998. Health Econ.

[9] Xu K, Evans DB, Kawabata K (2003). Understanding household catastrophic health expenditure: a multi-country analysis.

[10] Xu K, Evans DB, Carrin G, Aguilar-Rivera AM, Musgrove P, Evans T (2007). Protecting households from catastrophic health spending. Health Aff (Millwood).

[11] Pradhan M, Prescott N (2002). Social risk management options for medical care in Indonesia. Health Econ.

[12] O’Donnell O, van Doorslaer E, Rannan-Eliya RP, Somanathan A, Adhikari SR, Akkazieva B, Harbianto D, Garg CC, Hanvoravongchai P, Herrin AN (2008). Who pays for health care in Asia?. J Health Econ.

[13] Wagstaff A, van Doorslaer E, van der Burg H, Calonge S, Christiansen T, Citoni G, Gerdtham UG, Gerfin M, Gross L, Hakinnen U (1999). Equity in the finance of health care: some further international comparisons. J Health Econ.

[14] Ataguba JE, Akazili J, McIntyre D (2011). Socioeconomic-related health inequality in South Africa: evidence from General Household Surveys. Int J Equity Health.

[15] McIntyre D, Thiede M, Birch S (2009). Access as a policy-relevant concept in low- and middle-income countries. Health Econ Policy Law.

[16] Lin W, Liu GG, Chen G (2009). The Urban resident basic medical insurance: a landmark reform towards universal coverage in China. Health Econ.

[17] Tang S, Tao J, Bekedam H (2012). Controlling cost escalation of healthcare: making universal health coverage sustainable in China. BMC Public Health.

[18] Information Office of the State Council of the People’s Republic of China. Progress in China’s Human Rights in 2013. 2014 [http://english.cri.cn/6909/2014/05/26/2361s828338_15.htm]

[19] Zhang C, Li M, Wu C, Peng Z, Zhang Z, Li T (2011). Analyze on declining compensation proportion of New Rural Cooperative Medical Care. Chinese Primary Health Care.

[20] China’s health care reform. [http://www.fmprc.gov.cn/ce/cede/det/dshd/t787403.htm]

[21] Konings P, Harper S, Lynch J, Hosseinpoor AR, Berkvens D, Lorant V, Geckova A, Speybroeck N (2010). Analysis of socioeconomic health inequalities using the concentration index. Int J Public Health.

[22] Sun X, Jackson S, Carmichael G, Sleigh AC (2009). Catastrophic medical payment and financial protection in rural China: evidence from the New Cooperative Medical Scheme in Shandong Province. Health Econ.

[23] Gao J, Tang S, Tolhurst R, Rao K (2001). Changing access to health services in urban China: implications for equity. Health Policy Plan.

[24] Tang S, Li X, Wu Z (2006). Rising cesarean delivery rate in primiparous women in urban China: evidence from three nationwide household health surveys. Am J Obstet Gynecol.

[25] Pardeshi GS (2010). Age heaping and accuracy of age data collected during a community survey in the yavatmal district, maharashtra. Indian J Community Med.

[26] Gotsadze G, Zoidze A, Rukhadze N (2009). Household catastrophic health expenditure: evidence from Georgia and its policy implications. BMC Health Serv Res.

[27] Van Doorslaer E, Koolman X (2004). Explaining the differences in income-related health inequalities across European countries. Health Econ.

[28] Kakwani N, Wagstaff A, Van Doorslaer E (1997). Socioeconomic inequalities in health: measurement, computation, and statistical inference. J Econometrics.

[29] Wagstaff A (2005). The bounds of the concentration index when the variable of interest is binary, with an application to immunization. Health Econ.

[30] Wagstaff A (2000). Measuring and testing for inequality in delivery of health care. J Hum Resour.

[31] Gravelle H (2003). Measuring income related inequality in health: standardization and the partial concentration index. Health Econ.

[32] Yiengprugsawan V, Lim LL, Carmichael GA, Sidorenko A, Sleigh AC (2007). Measuring and decomposing inequity in self-reported morbidity and self-assessed health in Thailand. Int J Equity Health.

[33] Liu X, Gao W, Yan H (2014). Measuring and decomposing the inequality of maternal health services utilization in western rural China. BMC Health Serv Res.

[34] Hosseinpoor AR, Van Doorslaer E, Speybroeck N, Naghavi M, Mohammad K, Majdzadeh R, Delavar B, Jamshidi H, Vega J (2006). Decomposing socioeconomic inequality in infant mortality in Iran. Int J Epidemiol.

[35] Wang H, Zhang L, Hsiao W (2006). Ill health and its potential influence on household consumptions in rural China. Health Policy.

[36] Kavosi Z, Rashidian A, Pourreza A, Majdzadeh R, Pourmalek F, Hosseinpour AR, Mohammad K, Arab M (2012). Inequality in household catastrophic health care expenditure in a low-income society of Iran. Health Policy Plan.

[37] Arsenault C, Fournier P, Philibert A, Sissoko K, Coulibaly A, Tourigny C, Traore M, Dumont A (2013). Emergency obstetric care in Mali: catastrophic spending and its impoverishing effects on households. Bull World Health Organ.

[38] Wenqi F, Baoyu L, Qinghua Z, Guoxiang L (2014). Analysis on catastrophic health expenditure in rural households of Heilongjiang. Chinese Health Economics.

[39] Lagomarsino G, Garabrant A, Adyas A, Muga R, Otoo N (2012). Moving towards universal health coverage: health insurance reforms in nine developing countries in Africa and Asia. Lancet.

[40] Abolhallaje M, Hasani S, Bastani P, Ramezanian M, Kazemian M (2013). Determinants of catastrophic health expenditure in Iran. Iran J Public Health.

[41] Yan J, Yan Y, Hao N, Yang J, Gao J, Li Q, Wang Y, Lai S (2012). Empirical studies on the relief effect of catastrophic health expenditure under three basic medical schemes. Chinese Health Economics.

[42] Health Department of Shaanxi Province. The expanded program on immunization has achieved great improvement in Shaanxi Province. 2011 [http://www.shaanxi.gov.cn/0/1/9/39/100026.htm]

[43] Van Doorslaer E, Masseria C, Koolman X (2006). Inequalities in access to medical care by income in developed countries. CMAJ.

